# Impact of 5-HT_3_ receptor antagonists on chemotherapy-induced nausea and vomiting: a retrospective cohort study

**DOI:** 10.1186/1472-6963-12-215

**Published:** 2012-07-23

**Authors:** Swu-Jane Lin, Hind T Hatoum, Deborah Buchner, David Cox, Sanjeev Balu

**Affiliations:** 1 University of Illinois at Chicago, South Wood Street, Chicago, IL, USA; 2 Hind T. Hatoum & Company, Wilmot Avenue, Chicago, IL, USA; 3 Eisai, Inc, 100 Tice Blvd, Woodcliff Lake, NJ, USA

## Abstract

**Background:**

1^st^ generation 5-hydroxytryptamine receptor antagonists (5-HT_3_ RAs), and palonosetron, a 2^nd^ generation 5-HT_3_ RA, are indicated for the prevention of chemotherapy (CT)-induced nausea and vomiting (CINV) associated with moderately (MEC) and highly emetogenic CT agents (HEC). This study explores the impact of step therapy policies requiring use of an older 5-HT_3_ RA before palonosetron on risk of CINV associated with hospital or emergency department (ED) admissions.

**Methods:**

Patients who received cyclophosphamide post breast cancer (BC) surgery or who were diagnosed with lung cancer on carboplatin (LC-carboplatin) or cisplatin (LC-cisplatin) were selected from PharMetrics’ (IMS LifeLink) claims dataset (2005-2008). Patients were followed for 6 months from initial CT administration for CINV events identified through ICD-9-CM codes. Patients were grouped into those initiated with older, generic 5-HT_3_ RAs (ondansetron, granisetron, and dolasetron) and those initiated and maintained on palonosetron throughout study follow-up. CINV events and CINV days were analyzed using multivariate regressions controlling for demographic and clinical variables.

**Results:**

Eligible patients numbered 3,606 in BC, 4,497 in LC-carboplatin and 1,154 in LC-cisplatin cohorts, with 52%, 40%, and 34% in the palonosetron group, respectively. There was no significant difference between the two 5-HT_3_ RA groups in age or Charlson Comorbidity Index among the two MEC cohorts (BC and LC-carboplatin). Among the LC-cisplatin cohort, palonosetron users were older with more males than the older 5-HT_3_ RA group (age: 60.1 vs. 61.3; males, 66.9% vs. 56.9%). Compared to the older 5-HT_3_ RAs, the palonosetron groups incurred 22%-51% fewer 5-HT_3_ RA pharmacy claims, had fewer patients with CINV events (3.5% vs. 5.5% in BC, 9.5% vs. 12.8% in LC-carboplatin, 16.4% vs. 21.7% in LC-cisplatin), and had lower risk for CINV events (odds ratios 0.62, 0.71, or 0.71, respectively; p < 0.05). The BC and LC-carboplatin palonosetron groups experienced 50% and 30% fewer CINV days than the generic 5-HT_3_ RA group (p < 0.05).

**Conclusions:**

Patients with breast or lung cancer initiated and maintained on palonosetron were at significantly lower risk for potentially costly CINV versus those on older 5-HT_3_ RAs. Further studies on impact of step therapy policy are warranted in order to minimize the clinical and economic burden of CINV.

## Background

Antiemetics including the 5-hydroxytryptamine receptor antagonists (5-HT_3_ RAs) have been used as prophylaxis against chemotherapy-induced nausea and vomiting (CINV) in patients receiving moderately (MEC) or highly emetogenic (HEC) chemotherapy [1,2]. Palonosetron (Aloxi^®^) is a newer 5-HT_3_ RA, approved in the US in 2003 for antiemetic prophylaxis in patients with cancer receiving MEC or HEC.

Palonosetron is a potent and highly selective 5-HT_3_ RA with a strong binding affinity and a longer plasma-elimination half-life relative to the older 5-HT_3_ RAs [3]. Among the 5-HT_3_ RAs, palonosetron is unique in that it exhibits allosteric interactions, triggers receptor internalization, differentially inhibits “crosstalk” between NK-1 receptors and 5-HT_3_ signaling pathways, with prolonged inhibition of receptor function [4,5]. In four phase III trials, two in MEC and two in HEC, palonosetron was reported to have improved efficacy relative to the older 5-HT_3_ RAs in the prevention of nausea and vomiting associated with initial and repeat courses of MEC or HEC [2,6-8]. The available pre-clinical and clinical evidence suggests that the control of CINV in the delayed phase is a true pharmacological effect reflecting the unique properties of palonosetron rather than a carryover effect from better control during the acute phase [9].

The step-wise use of medications commonly referred to as step-therapy is a pharmacy administration interventional tool employed to encourage the use of purportedly therapeutically equivalent, but lower-cost medications from the same or different therapeutic classes. Studies of step therapy have reported mixed results in terms of step therapy’s ability to control drug and total medical expenditures. In fact, several of these studies have reported that the initial cost savings of implementing step therapy programs diminished over time [10-13], resulting in a negative impact on medical utilization and associated costs [10,11,13]. Moreover, a recent commissioned report by the Academy of Managed Care Pharmacy (AMCP), a professional association of pharmacists and other health care practitioners who practice pharmaceutical care in managed health care environments[14], concluded that there is a paucity of research examining the effects of step therapy on clinical and humanistic outcomes [15].

Because palonosetron is a newer 5-HT_3_ RA antiemetic, oncology practices and managed care institutions may consider some form of step therapy, whereby an older and generic 5-HT_3_ RA would be prescribed as initial therapy for CINV prophylaxis, reserving palonosetron for second-line prophylaxis or as rescue medication as a means to contain costs. The current study assesses the use of a step therapy-like approach to guide the prescribing choice between palonosetron and the older 5-HT_3_ RAs as to its potential clinical and economic impact. The objectives of the study were to: 1) identify patterns of therapy with 5-HT_3_ RA-based antiemetic regimens indicative of step therapy approaches in patients newly treated with MEC or HEC, 2) explore the characteristics of patients and health plans associated with 5-HT_3_ RA step therapy prescribing patterns, and 3) evaluate the impact of a step therapy approach of initiating patients on an older 5-HT_3_ RA before using palonosetron with regard to differences in the incidence of hospital/emergency department (ED) associated CINV events.

## Methods

The study population was selected from the PharMetrics (IMS LifeLink) claims database, which compiles integrated managed care pharmacy and medical claims of over 2 billion inpatient and outpatient transactions from 60 million members in the United States. The database is longitudinal, going back as far as 1995, and contains information on paid claims for drugs (drug name, quantity dispensed, length of supply), medical diagnoses, and procedures, and demographics of each patient. Prescription drugs are coded with the National Drug Code (NDC) from the US Food and Drug Administration. Diagnoses are coded using the International Classification of Diseases-Clinical Modification, 9th Revision (ICD-9-CM), and procedures are coded using the Current Procedural Terminology-4 (CPT) and Healthcare Common Procedure Coding System (HCPCS). This database has been used for many previous studies [16].

The study included patients diagnosed with breast cancer (BC) who received adjuvant chemotherapy with cyclophosphamide within four months after surgery, along with patients diagnosed with lung cancer (LC) who received carboplatin (LC-carboplatin), and patients diagnosed with lung cancer (LC) who received cisplatin-based chemotherapy (LC-cisplatin). The study time frame was from January 1, 2005 through June 2008. Based on the doses used and the published literature [17], the BC and the LC-carboplatin cohorts were considered MEC-treated, while the LC-cisplatin cohort was considered HEC-treated. The study index date was defined as the first date of BC diagnosis for the BC cohort or the first date of chemotherapy with carboplatin/cisplatin for the LC cohorts. The index chemotherapy was the first chemotherapy treatment cycle and the index chemotherapy date was marked as the first day of the index chemotherapy.

Patients with BC were identified using the ICD-9-CM codes (174.xx). Patients with LC were identified by ICD-9-CM codes of 162.xx. NDC and HCPCS codes were used to identify oral and injectable chemotherapies, respectively. Selected patients were ≥18 years of age having records of claims for ≥6 months prior to study index date. Additional inclusion criteria included patients not having prior history of vomiting, nausea, or dehydration identified by ICD-9-CM codes during the 6 months preceding the index chemotherapy and patients having a ≥6 months follow-up period after the index chemotherapy. Study duration was defined as six months from the index chemotherapy date. Patients who received cyclophosphamide, carboplatin, or cisplatin within the six months prior to index chemotherapy were excluded. Antiemetics investigated in this study were dolasetron, granisetron, ondansetron, and palonosetron. The concomitant use of either aprepitant or dexamethasone was also included within the analytic comparison groups. Injectable antiemetics were identified from the HCPCS codes for inpatient and outpatient claims while those administered orally were identified through appropriate NDCs in the prescription drug claims file. Based on the prescribing patterns of 5-HT_3_ RAs, patients were classified in two groups: the palonosetron group consisting of patients initiated with palonosetron on the index CT date and maintained on it for the study duration, and the older 5-HT_3_ RA group, who were initiated with one of the first generation 5-HT_3_ RAs, and then were either maintained on the initial agent or alternated between any 5-HT_3_ RAs (including palonosetron) in the subsequent 6-month study duration. The use of aprepitant and dexamethasone was assessed in both study groups.

Severity of patient conditions identified through comorbidities during the 6-month baseline period before the study index date were summarized using the Charlson Comorbidity Index (CCI), an aggregate measure with higher scores denoting greater disease burden [18,19]. Health insurance related variables included type of plan (health maintenance organization, indemnity, preferred provider organization, point of service, others), type of payer (commercial, public, self-insured), and pharmacy benefit plans.

CINV events were extracted using both paid and filed claims with ICD-9-CM codes for nausea, vomiting, and/or dehydration (787.0, 787.01, 787.02, 787.03, 276.5, 276.50, 276.51, and 276.52). CINV events associated with hospital/ED admissions were selected. The identification of practice setting (e.g., hospital, ED, physician office) where a CINV event occurred was based on an algorithm that employed several variables such as provider specialties, place of service, and HCPCS codes. Total days on cyclophosphamide, carboplatin, or cisplatin within the study duration were also calculated for the three study cohorts.

## Statistical analyses

Descriptive statistics are reported for all variables used in this study. For continuous variables, t-tests were performed to compare palonosetron group to the older 5-HT_3_ RA group. Nonparametric Wilcoxon tests were used to compare days with hospitalization/ED related CINV events and for categorical variables, chi-square tests were performed to compare between the groups. Multivariate logistic regression models and Poisson regression models were performed separately for the three cohorts. Logistic regression models, through backward elimination, investigated the risk of any occurrence of hospital/ED associated CINV events, while Poisson regression models examined the number of days with hospital/ED associated CINV events. Both regression models controlled for age, CCI, gender (lung cancer cohorts only), and days on CT with cyclophosphamide, carboplatin, or cisplatin. A negative binomial distribution was used to control for over-dispersion in Poisson regressions. All statistical analyses were carried out with SAS 9.0 statistical software (SAS Institute, Inc, Cary, NC, USA).

## Results

### Demographic characteristics of the study cohorts

A total of 3,606 patients with BC, 4,497 patients in LC-carboplatin, and 1,154 in LC-cisplatin cohorts were included. Among the selected study population, 1,864 (52 %) patients with BC, 1,806 (40%) patients in the LC-carboplatin and 390 (34%) patients in the LC-cisplatin cohorts were in the palonosetron group. The mean (SD) age was 53.7 (9.8) years for the BC cohort, 64.9 (10.2) years for the LC-carboplatin, and 60.5 (9.8) years for the LC-cisplatin cohorts. Calculated CCIs at baseline were 0.5 (1.0), 6.8 (3.2), and 6.5 (3.3) in BC, the LC-carboplatin, and the LC-cisplatin cohorts, respectively.

There were no significant differences between the older 5-HT_3_ RA and the palonosetron groups in age and CCI in the BC and LC-carboplatin cohorts. In the LC-cisplatin cohort, the palonosetron group was significantly older (mean, 61.3 versus 60.1 years, p < 0.05), and had fewer females (33.3% versus 43.1%, p < 0.005) than the older 5-HT_3_ RA group (Table 1).

**Table 1:** Patients and provider characteristics

	**Breast cancer**				**Carboplatin-treated lung cancer**				**Cisplatin-treated lung cancer**			
	**All**	**Older 5-HT**_**3**_**RAs**	**Palonosetron**	**P**	**All**	**Older 5-HT**_**3**_**RAs**	**Palonosetron**	**P**	**All**	**Older 5-HT**_**3**_**RAs**	**Palonosetron**	**P**
N	3,606	1,742	1,864		4,497	2,691	1,806		1,154	764	390	
**Age mean (SD)**	53.7 (9.8)	53.7 (9.7)	53.7 (10.0)	NS	64.9 (10.2)	64.77 (10.2)	65.03 (10.3)	NS	60.5 (9.8)	60.1 (9.8)	61.3 (9.8)	0.0476
**CCI mean (SD)**	0.5 (1.0)	0.5 (1.1)	0.5 (1.0)	NS	6.8 (3.2)	6.87 (3.2)	6.71 (3.2)	NS	6.5 (3.3)	6.6 (3.3)	6.3 (3.2)	NS
**Female, N (%)**	-	-	-	-	2,080 (46.3)	1,258 (46.8)	822 (45.5)	NS	459 (39.8)	329 (43.1)	130 (33.3)	0.0014
**Year of 5-HT**_**3**_**RA initiated**				<0.0001				<0.0001				NS
2005	1,428 (39.6)	753 (43.3)	675 (36.2)		1,562 (34.7)	990 (36.8)	572 (31.7)		348 (30.2)	247 (32.3)	101 (25.9)	
2006	1,537 (42.6)	720 (41.3)	817 (43.8)		1,480 (32.9)	857 (31.9)	623 (34.5)		394 (34.1)	263 (34.4)	131 (33.6)	
2007	520 (14.4)	223 (12.8)	297 (15.9)		1,140 (25.4)	687 (25.5)	453 (25.1)		315 (27.3)	194 (25.4)	121 (31.0)	
2008	121 (3.4)	46 (2.6)	75 (4.0)		315 (7.0)	157 (5.8)	158 (8.8)		97 (8.4)	60 (7.9)	37 (9.5)	

P: P value; CCI: Charlson Comorbidity Index; 5-HT_3_ RAs: 5-hydroxytryptamine receptor antagonists.

### Health insurance and payer characteristics

More than 90% of the patients in the breast cancer cohort and 80% of the patients in the lung cancer cohorts were insured by commercial payers, most likely reflecting the data source. There were no statistically significant differences in the payer mix between the two comparison groups for all three cancer cohorts (Table 2). However, there were significant differences found in the distribution of health plan type between the older 5-HT_3_ RA and the palonosetron groups. More specifically, the proportion of patients enrolled in health maintenance organizations (HMO) was lower in the palonosetron group compared to the older 5-HT_3_ RA group (20.3% vs. 24.9% in the BC cohort, and 18.7% vs. 27.3% in LC-carboplatin cohort, both at p < 0.0001). Similarly, the palonosetron group, for both the BC and LC-carboplatin cohorts, was less likely to have pharmacy benefit type plan. The proportion of patients with pharmacy benefits was 63.0% vs. 68.8% in the BC cohort and 57.6% vs. 65.7% in the LC-carboplatin cohort, p < 0.001. No significant differences were found in the LC-cisplatin cohort regarding health plan type or in having a pharmacy benefit.

**Table 2:** Health insurance characteristics

	**Breast cancer**				**Carboplatin-treated lung cancer**				**Cisplatin-treated lung cancer**			
	**All**	**Older 5-HT**_**3**_**RAs**	**Palonosetron**	**P**	**All**	**Older 5-HT**_**3**_**RAs**	**Palonosetron**	**P**	**All**	**Older 5-HT**_**3**_**RAs**	**Palonosetron**	**P**
N	3,606	1,742	1,864		4,497	2,691	1,806		1,154	764	390	
**Type of payer (%)**				NS				NS				NS
Commercial	3,321 (92.1)	1,592 (91.4)	1,729 (92.8)		3,632 (80.8)	2,149 (79.9)	1,483 (82.1)		971 (84.1)	638 (83.5)	333 (85.4)	
Public	167 (4.6)	87 (5.0)	80 (4.3)		715 (15.9)	448 (16.7)	267 (14.8)		121 (10.5)	81 (10.6)	40 (10.3)	
Self-insured	100 (2.8)	54 (3.1)	46 (2.5)		134 (3.0)	84 (3.1)	50 (2.8)		55 (4.8)	39 (5.1)	16 (4.1)	
Unknown	18 (0.5)	9 (0.5)	9 (0.5)		16 (0.4)	10 (0.4)	6 (0.3)		7 (0.6)	6 (0.8)	1 (0.3)	
**Type of plan (%)**				<0.0001				<0.0001				NS
HMO	813 (22.6)	434 (24.9)	379 (20.3)		1,071 (23.8)	734 (27.3)	337 (18.7)		272 (23.6)	182 (23.8)	90 (23.1)	
Indemnity	320 (8.9)	136 (7.8)	184 (9.9)		854 (19.0)	499 (18.5)	355 (19.7)		170 (14.7)	112 (14.7)	58 (14.9)	
PPO	1,742 (48.3)	792 (45.5)	950 (51.0)		1,982 (44.1)	1,114 (41.4)	868 (48.1)		551 (47.8)	360 (47.1)	191 (49.0)	
POS	556 (15.4)	316 (18.1)	240 (12.9)		462 (10.3)	278 (10.3)	184 (10.2)		120 (10.4)	77 (10.1)	43 (11.0)	
Other	175 (4.9)	64 (3.7)	111 (6.0)		128 (2.9)	66 (2.5)	62 (3.4)		41 (3.6)	33 (4.3)	8 (2.1)	
**Pharmacy benefit (%)**				0.0008				<0.0001				NS
No	700 (19.4)	300 (17.2)	400 (21.5)		1,229 (27.3)	656 (24.4)	573 (31.7)		246 (21.3)	151 (19.8)	95 (24.4)	
Yes	2,372 (65.8)	1,198 (68.8)	1,174 (63.0)		2,809 (62.5)	1,769 (65.7)	1,040 (57.6)		761 (65.9)	518 (67.8)	243 (62.3)	
Unknown	534 (14.8)	244 (14.0)	290 (15.6)		459 (10.2)	266 (9.9)	193 (10.7)		147 (12.7)	95 (12.4)	52 (13.3)	

HMO: health maintenance organization, PPO: preferred provider organization, POS: point of service, pts: patients.

### Chemotherapy and antiemetic claims

During the 6-month study follow-up, the palonosetron group in the BC and LC-cisplatin cohorts had significantly fewer cyclophosphamide or cisplatin treatment days, as compared to their older 5-HT_3_ RA groups (4.2 versus 4.3 days in BC, p = 0.0297; and 4.9 versus 6.3 days in the LC-cisplatin, p < 0.0001, Table 3). Treatment days with CT were not significantly different between the two comparison groups in the LC-carboplatin cohort (6.1 versus 6.2 days). The palonosetron group in all three study cohorts had used significantly less antiemetics than that used by the older 5-HT_3_ RA groups. In fact, the mean number of 5-HT_3_ RA claims in the BC cohort was 6.2 in the palonosetron group versus 7.9 in the older 5-HT_3_ RA group. Similarly, the mean claims were 7.7 and 10.3 for the two comparison groups in the LC-carboplatin cohort and were 6.4 and 13.1 in the LC-cisplatin cohort (all P < 0.0001). When the utilization of aprepitant and dexamethasone were included, the total number of antiemetic claims in the palonosetron groups remained significantly lower than the total claims for the older 5-HT_3_ RA groups among all three cancer cohort (all p < 0.0001). On average, the palonosetron group had 22-51% fewer 5-HT_3_ RA claims, and 16-44% fewer total antiemetic claims depending on whether the antiemtic regimens were associated with MEC or HEC.

**Table 3:** Chemotherapy, antiemetic prophylaxis and CINV outcomes

	**Breast cancer**				**Carboplatin-treated lung cancer**				**Cisplatin-treated lung cancer**			
	**All**	**Older 5-HT**_**3**_**RAs**	**Palonosetron**	P*	**All**	**Older 5-HT**_**3**_**RAs**	**Palonosetron**	**P**	**All**	**Older 5-HT**_**3**_**RAs**	**Palonosetron**	**P**
N	3,606	1,742	186		4,497	2,691	1,806		1,154	764	390	
CT tx days, mean (SD)	4.2 (1.2)	4.3 (1.3)	4.2 (1.2)	0.0297	6.1 (3.7)	6.2 (3.6)	6.1 (3.6)	NS	5.8 (4.3)	6.3 (4.7)	4.9 (3.2)	<0.0001
All antiemetic claims	9.1 (5.1)	9.9 (5.5)	8.3 (4.6)	<0.0001	10.2 (6.7)	11.4 (7.2)	8.5 (5.4)	<0.0001	13.0 (8.0)	15.3 (8.0)	8.5 (5.6)	<0.0001
All 5-HT_3_ RA claims	7.0 (3.9)	7.9 (4.2)	6.2 (3.4)	<0.0001	9.2 (6.0)	10.3 (6.4)	7.7 (4.9)	<0.0001	10.9 (6.9)	13.1 (6.8)	6.4 (4.4)	<0.0001
Days with hospital/ED associated CINV events	0.1 (0.7)	0.1 (0.9)	0.1 (0.4)	0.0052	0.3 (1.3)	0.3 (1.4)	0.2 (1.2)	0.0004	0.6 (2.0)	0.7 (2.1)	0.6 (1.9)	0.0499
Pts with > =1 hospital/ED associated CINV events, N (%)	161 (4.5)	95 (5.5)	66 (3.5)	0.0055	516 (11.5)	345 (12.8)	171 (9.5)	0.0005	230 (19.9)	166 (21.7)	64 (16.4)	0.0324

CT: chemotherapy, Tx: treatment, ED: emergency department, pts: patients.

P values were based on t-tests, except for days and proportions of patients with hospital/ED associated CINV events, where the nonparametric Wilcoxon and Chi-square tests were used, respectively.

### Unadjusted risk for CINV

Comparing the unadjusted risk for CINV, there were fewer patients in the palonosetron groups with one or more hospital/ED associated CINV events than those in the older 5-HT_3_ RA groups. The percentage of patients who experienced CINV was 3.5% in the palonosetron group and 5.5% in the older 5-HT_3_ RA groups for the BC cohort (p = 0.0055). Similarly, the proportions were 9.5% vs. 12.8% (p = 0.0005) in the LC-carboplatin and 16.4% vs. 21.7% (p = 0.0324) in the LC-cisplatin cohorts, respectively. Furthermore, patients in the palonosetron group had significantly fewer days with hospital/ED associated CINV events than patients in the older, generic 5-HT_3_ RA groups with means of 0.06 vs. 0.11 days among BC patients, 0.24 versus 0.34 in the LC-carboplatin and 0.57 versus 0.67 in the LC-cisplatin cohorts, all p < 0.05; Table 3.

### Adjusted risk for CINV

In the BC cohort, logistic regression models with backward elimination using hospital/ED associated CINV events as the outcome variable, and controlling for age, CCI, and cyclophosphamide treatment days, found the palonosetron group to have significantly lower risk (38%) of outcome [Odds Ratio (OR) = 0.62, p = 0.0035; Table 3]. In addition, a higher CCI score was found to significantly increase the risk of outcome, while more CT days was associated with a reduced risk (p < 0.05, Table 4). In the LC-carboplatin cohort, the palonosetron group experienced 29% lower risk of hospital/ED associated CINV events than the older 5-HT_3_ RA group (OR = 0.71, p = 0.0006). The model with LC-carboplatin cohort found that a higher CCI score increased the risk of outcome, while females and patients with more carboplatin treatment days experienced lower risk (p < 0.05; Table 4). Similarly, the palonosetron group in the LC-cisplatin cohort experienced a 29% reduced risk of hospital/ED associated CINV events as compared to the older 5-HT_3_ RA group (OR = 0.71, p = 0.0330, Table 4).

**Table 4:** Results of the logistic regression models on the risk of hospital/ED associated CINV events

**Parameter**	**Breast cancer (N = 3606)**		**Carboplatin-treated Lung cancer (N = 4497)**		**Cisplatin-treated lung cancer (N = 1154)**	
	**OR (95% CI)**	**P value**	**OR (95% CI)**	**P value**	**OR (95% CI)**	**P value**
age	NS	NS	NS	NS	NS	NS
CCI	1.247 (1.122-1.387)	<.0001	1.042 (1.012-1.073)	0.0052	NS	NS
Gender (male as reference)						
Female	NA	NA	0.817 (0.678-0.985)	0.0338	NS	NS
Days with cyclophosphamide (in breast cancer) or carboplatin or cisplatin (in lung cancer) treatment	0.719 (0.619-0.836)	<.0001	0.942 (0.915-0.969)	<.0001	NS	NS
Antiemetic regimen (older 5-HT_3_ RAs as reference)						
Palonosetron	0.618 (0.447-0.854)	0.0035	0.712 (0.586-0.864)	0.0006	0.707 (0.514-0.972)	0.0330

NA: not applicable, NS: Not significant.

In both the BC and the LC-carboplatin cohorts, Poisson regression analyses with days of hospital/ED associated CINV events as the dependent variable found that the palonosetron group incurred significantly fewer CINV days as compared to the older 5-HT_3_ RA groups after controlling for age, CCI score, and CT treatment days. In the BC cohort, the total days with hospital/ED associated CINV events in the palonosetron group was almost half as much (52.6%) of those incurred by patients in the older 5-HT_3_ RA group (95% CI, 35.4%-78.1%, p = 0.0015, Table 5). Similar to the findings in the logistic regression, a higher CCI score also significantly increased CINV days while more CT days was associated with a reduced number of CINV days (p < 0.05) in the BC cohort. In the LC-carboplatin cohort, the total CINV days experienced by the palonosetron group was 70.9% of those incurred by the older 5-HT_3_ RA group while a higher CCI score increased and more chemotherapy days reduced CINV days (p < 0.05). In the LC-cisplatin cohort, the model did not show a significant difference between the two 5-HT_3_ RA groups in CINV days, although the negative coefficient for the palonosetron group denotes a decrease in CINV days (Table 5).

**Table 5:** Results of the Poisson regression models on days of hospital/ED associated CINV events

**Parameters**	**Breast cancer (N = 3606)**		**Carboplatin-treated lung cancer (N = 4497)**		**Cisplatin-treated lung cancer (N = 1154)**	
	**Regression coefficient (95% CI)**	**P value**	**Regression coefficient (95% CI)**	**P value**	**Regression coefficient (95% CI)**	**P value**
Age	-0.0032 (-0.0219; 0.0155)	0.7354	-0.0008 (-0.0120; 0.0105)	0.8961	-0.0149 (-0.0325; 0.0027)	0.0968
CCI	0.2529 (0.0572; 0.4486)	0.0113	0.0448 (0.0087; 0.0810)	0.0151	0.0747 (0.0182; 0.1312)	0.0096
Gender (male as reference)						
Female	NA		-0.1815 (-0.4202; 0.0573)	0.1363	-0.0132 (-0.3975; 0.3711)	0.9463
Days with cyclophosphamide (in breast cancer) or carboplatin (in lung cancer) treatment	-0.3800 (-0.5600; -0.2000)	<.0001	-0.0483 (-0.0848; -0.0118)	0.0095	0.0113 (-0.0263; 0.0488)	0.5561
Antiemetic regimen (older 5-HT_3_ RAs as reference)						
Palonosetron	-0.6431 (-1.0391; -0.2471)*	0.0015	-0.3436 (-0.5871; -0.1001)*	0.0057	-0.0373 (-0.4456; 0.3711)	0.8580

* In Poisson regression, the exponential of coefficient indicates the ratio of the comparison groups in outcome. The exponential of the coefficient of antiemetic regimen (-0.6431) was 0.526 in the BC cohort, indicating the average CINV-hospitalization/ED days of palonosetron group was about 52.6% of that of the comparison group (older 5-HT_3_ RAs). The exponential of the coefficient of antiemetic regimen (-0.3436) was 0.709 in the LC-carboplatin cohort, indicating the average CINV-hospitalization/ED days of palonosetron group was about 70.9% of that of the comparison group (older 5-HT_3_ RAs).

## Discussion

There are relatively few studies in the literature that have evaluated the implications of pharmacy administration step therapy policies on clinical and economic outcomes [15]. Specifying two scenarios, one deals with initiating antiemetic therapy on palonosetron, a second generation 5-HT_3_ RA, and maintaining this regimen throughout the patient’s chemotherapy experience versus another dealing with initiating antiemetic therapy with an older 5-HT_3_ RA, regardless of whether palonosetron or an older 5-HT_3_ RA was used subsequently, a natural experiment was formulated utilizing real-world claims data. Comparing the subsequent CINV events associated with hospital/ED admissions of patients who were initiated and maintained on the respective 5-HT_3_ RA-based regimens supported an indirect evaluation of a step therapy approach and formed the basis of this investigation. The analysis focused on two MEC cohorts, patients with breast cancer receiving a cyclophosphamide-based regimen and patients with lung cancer receiving carboplatin treatment, and one HEC cohort of patients with lung cancer on cisplatin treatment. In addition to the primary study end point of CINV-associated hospital/ED events, a secondary outcome addressed days with CINV events associated with hospitalizations or ED admissions. The focus on hospital/ED associated CINV was to capture the most costly healthcare resources consumed resulting from uncontrolled CINV.

Among the breast cancer, lung cancer-carboplatin and lung cancer-cisplatin cohorts, 48%, 60%, and 66% of the patients, respectively, were started with an older 5-HT_3_ RA as the initial antiemetic to prevent CINV. The study showed that the risk of one or more hospital/ED associated CINV events was significantly lower in the palonosetron groups as compared to the older, generic 5-HT_3_ RA groups, with reductions between 30-40%, depending on the emetogenicity of the chemotherapy. In multiple regression analyses, and for all three study cohorts, the palonosetron groups experienced lower risk in the form of reduced CINV events, fewer days with hospital/ED events, fewer 5-HT_3_ RA claims, and fewer total antiemetic claims than those incurred by the older 5-HT_3_ RA treated groups.

The correlation between palonosetron prophylaxis and clinical outcomes found in our retrospective analysis corroborates data obtained from clinical trials. In both settings, palonosetron was associated with a significantly reduced risk of developing CINV events as compared with the older 5-HT_3_ RAs. Additionally, a recently published study that compared palonosetron and ondansetron using medical record review found that in patients with gynecological cancers treated with cisplatin, palonosetron was associated with a trend of a lower risk of CINV-related hospital readmission than ondansetron [20].

Difference in index date for patients with breast cancer from that used in patients with lung cancer reflects the nature of the two clinical conditions. Since patients with breast cancer were included contingent upon having received surgical interventions with each patient having different time interval from initial diagnosis to surgical intervention and initiation of chemotherapy, an index date with time of diagnosis would consistently depict the patients’ clinical severity of this study cohort. Despite differences in the index dates among the three cancer cohorts, the impact on outcome would be neglible, because all the comparisons made were between groups within the same cohort instead of between cohorts.

A recent publication summarized research on the ability of interventions to contain drug expenditures in managed care settings over the past 10 years [15]. A total of 63 studies were identified, with seven focusing on step therapy type interventions. The report found that in some instances, savings in drug expenditures through step therapy are associated with an increase in total plan spend. The authors suggested that additional research was needed, especially with respect to the impact of these types of interventions on clinical and humanistic outcomes [15]. This study adds to the current body of knowledge by focusing on the clinical impact of step therapy as it relates to the 5-HT_3_ RA antiemetic drug class in a particularly vulnerable patient population.

The palonosetron groups in two of the three cohorts were less likely to have pharmacy benefits. We have reported significance in terms of less antiemetic use in these groups, however due to the nature of claims data, it is unclear whether or not some additional prescriptions were actually written, but not filled by the patients. As such, it is possible that patients with lower socioeconomic status (SES) may not have been adequately studied. This limitation is relevant to the extent that lower SES, independent of taking or not taking palonosetron, may explain some of the differences in the CINV experience. Additionally, it may be possible to infer that providers may have prescribed differently if insurance companies provided coverage for the otherwise not considered anti-emetic alternatives.

In the current study, all older 5-HT_3_ RAs were combined into one group, in part to simplify the study design, but also based on reported findings that patients on HEC who were treated with dolasetron, granisetron, or ondansetron, demonstrated no significant difference in antiemetic efficacy. [21]. Fewer chemotherapy treatment days, in and by itself, was associated with higher risk for CINV experience, because of the higher chemotherapy dose used in a shorter time frame. We reported that in the palonosetron treated group, there were both fewer days of chemotherapy and lower risk of CINV. This outcome would most likely be attributed to palonosetron treatment lowering the risk for CINV, in spite of the higher potential for the risk of CINV experience when a course of chemotherapy was given within a shorter time frame. Our results suggest that initiating and maintaining patients on palonosetron throughout all chemotherapy treatment cycles, rather than implementing a step therapy of initiating patients first on any one of the older 5-HT_3_ RAs, reduced the number of antiemetic medications and simultaneously achieved fewer hospital/ED associated CINV events.

### Limitations

This study had the inherent limitations associated with retrospective analyses. Patterns of 5-HT_3_ RA therapies were not solely responsible for the CINV outcomes reported. Additional important explanatory variables are not captured in claims data. These include race, alcohol consumption, cancer staging, and history of motion sickness, to name a few. Although efforts were made to delineate the relationship between timing of outcomes and 5-HT_3_ RA use, the temporal relationship between these two variables hinged on when providers submitted related claims.

Selection bias or confounding by indication could also be present in this study. The calculated CCI score and the employed multiple regressions may have adjusted for some but not all of the differences between the study groups. Finally, the claims dataset used in this study was mainly employer-based, thus potentially limiting the generalizability of the study findings since the elderly population would not have been adequately represented.

## Conclusion

Consistently across three separate cancer cohorts, initiating and maintaining patients on palonosetron was found associated with significant reduction in the risk and frequency of CINV events associated with hospitalization/ED admissions relative to what was achieved when older 5-HT_3_ RAs were used as the initial antiemetic. Results from this retrospective analysis suggest that step therapy approaches with respect to 5-HT_3_ RAs should take into consideration the implications for costly downstream CINV events.

## Competing interest

S. Lin is a consultant to Hind T. Hatoum & Company. H. T. Hatoum is a paid consultant to Eisai, Inc., the marketer of palonosetron in the United States and the sponsor of the study. Deborah Buchner. David Cox, and Sanjeev Balu are, or were employees of Eisai, Inc. The authors had full control of the all primary data and findings. All authors agree to allow the Journal to review the data if requested. Results are presented without any oversight or interference from the sponsor of the work.

## Authors' contributions

SJL performed the statistical analysis of data, and participated in the writing of the manuscript. HTH contributed to the study design, the interpretation of data and the writing of the manuscript. DB was involved in the conception of the study and its design, and in the writing of the manuscript, DC contributed to the interpretation of results, and to providing input into the important intellectual clinical content and to manuscript development, SB contributed to the design of the study, the interpretation of results, and in the writing of the manuscript . All authors gave final approval of the version submitted.

## Pre-publication history

The pre-publication history for this paper can be accessed here:

http://www.biomedcentral.com/1472-6963/12/215/prepub
